# Crystal structure of HERV-K envelope glycoprotein surface subunit

**DOI:** 10.1128/jvi.00195-26

**Published:** 2026-05-07

**Authors:** Nikos Nikolopoulos, Yorgo Modis

**Affiliations:** 1 Molecular Immunity Unit, Department of Medicine, MRC Laboratory of Molecular Biology, University of Cambridge, Cambridge, United Kingdom; 2 Cambridge Institute of Therapeutic Immunology & Infectious Disease (CITIID), Department of Medicine, University of Cambridge, Cambridge, United Kingdom; University Hospital Tübingen, Tübingen, Germany

**Keywords:** endogenous retrovirus, betaretrovirus, envelope glycoprotein, N-linked glycosylation, ligand binding site, X-ray crystallography, receptor-binding protein

## Abstract

The most recently acquired and transcriptionally active family of human endogenous retroviruses (HERVs) is HERV-K. Of the approximately 100 copies of HERV-K in our genome, many retain the potential to proliferate by retrotransposition, express viral proteins, and form functional virus particles. Aberrant expression of the HERV-K envelope glycoprotein (Env) has been associated with cancer and neurodegeneration. Autoantibodies against HERV-K Env have been found in patients with various autoimmune diseases. Here, we report the crystal structure of the Env surface subunit (SU) from HERV-K HML-2, determined at 2.25-Å resolution. The overall fold is somewhat similar to Syncytin-2 SU and distantly related to HIV-1 gp120. The structure contains five disulfides, four N-linked glycans, and two sulfate ions bound to a basic surface groove. Two extended loops form a surface for potential interactions with cell-surface receptors or other cellular factors. The structure also contains three steroid molecules bound to hydrophobic surface patches. This crystal structure provides a platform for future studies to map autoantigenic epitopes, identify small molecules that interfere with HERV-K activity, and extend our mechanistic understanding of retroviruses.

**IMPORTANCE:**

Eight percent to 15% of the human genome consists of endogenous retroviruses and other virus-derived elements inherited from ancestral viral infections. Many endogenous retroviruses from the HERV-K family retain the ability to proliferate across the genome and produce virus-like particles. Aberrant expression of the HERV-K envelope glycoprotein is associated with cancer, neurodegeneration, and autoimmune disease. Here, we report the crystal structure of the HERV-K envelope glycoprotein surface subunit. The structure provides an atomic-level view of the molecular components in HERV-K most likely to trigger autoimmune responses and identifies potential binding sites for drug-like molecules and cell-surface polysaccharides.

## INTRODUCTION

Endogenous retroviruses (ERVs) and other endogenous viral elements, relics of ancient germline infections, constitute up to 15% of the human genome (1, 2). Most human ERV (HERV) loci have been rendered nonfunctional by mutations, deletions, and recombination events, but HERV-K, belonging to a betaretrovirus-like group within the *Retroviridae* family and representing the most recent wave of endogenization, stands out as remarkably intact (3, 4). Many HERV-K, particularly the human MMTV-like family 2 (HML-2) proviruses, maintain full-length open reading frames (ORFs) for *gag*, *pol,* and *env* genes (5). Notably, reconstructed consensus HERV-K genomes can produce infectious virions *in vitro* (6, 7).

The HERV-K envelope glycoprotein (Env) has been implicated in both health and disease contexts. During early embryonic development, HERV-K Env is expressed on the surface of pluripotent stem cells, regulating their function via its association with CD98 and impacting neuronal differentiation (8). Although HERV-K transcription is regulated by DNA methylation in adult somatic cells (9), HERV-K (HML-2) proviruses are nevertheless broadly expressed in various healthy adult tissues, in particular in the cerebellum, testes and ovaries, and pituitary and thyroid glands (10, 11). Reactivation of HERV-K and expression of HERV-K Env is associated with a range of cancers, neurodegeneration, and autoimmune diseases (12–17). Expression of HERV-K Env has been reported in melanoma cells (12), breast cancer (13), and ovarian cancer (14). Autoantibodies against HERV-K Env have been reported in patients with systemic lupus erythematosus (SLE) (16), rheumatoid arthritis (RA) (17), and amyotrophic lateral sclerosis (16).

As a functional class I fusion protein, HERV-K Env follows a canonical structural organization and is synthesized as a precursor, which is cleaved into surface (SU) and transmembrane (TM) subunits (18). SU has been reported to engage with different host factors on the cell surface. Heparan sulfate glycosaminoglycan serves as a primary attachment factor (19). SU also interacts with the CD98 heavy chain, a component of amino acid transporter LAT1 (8, 20). However, the identity of the receptor that triggers viral membrane fusion following cellular attachment remains unknown. Here, we report the crystal structure of the receptor-binding unit (SU) of a human endogenous retrovirus at 2.25-Å resolution using a consensus sequence derived from ten HERV-K (HML-2) proviruses (7). Our crystal structure complements two cryo-EM reconstructions of the trimeric Env SU-TM (21, 22), published while this manuscript was in preparation, to provide a high-resolution view of the SU subunit, including glycosylation and disulfide bonds. In addition, we identify cholate derivatives bound within distinct SU pockets, and sulfate ions bound to a conserved negatively charged surface groove. These insights establish an atomic framework for dissecting HERV-K Env function and exploring strategies to counteract HERV-K Env-associated disease activity.

## RESULTS

### Crystal structure of HERV-K Env SU

Env SU (residues 111–430) of a consensus-sequence HERV-K construct, HERV-K_CON_ (7), was expressed in HEK293S GnTI^−^ cells to produce a uniform glycosylation pattern to facilitate glycoprotein crystallization. This consensus sequence was selected because it resembles the HERV-K (HML-2) progenitor that entered the human genome over a million years ago and retains the capacity to form infectious virions and lacks the inactivating mutations acquired by most current HERV-K (HML-2) variants (6, 7). HEK293S GnTI^−^ cells lack N-acetylglucosaminyltransferase I activity and accordingly secrete proteins with Man5GlcNAc2 (mannopentaose-di-(N-acetyl-D-glucosamine)) as the predominant N-linked glycosylation. The long N-terminal native signal peptide of HERV-K Env (residues 1–110) was replaced by the signal peptide-pro-peptide domain from human Trypsin-1 to enhance protein secretion (23). Substitution of cysteine at position 141 with alanine (C141A) abolished non-specific protein cross-linking and aggregation through aberrant disulfide bond formation. The abovementioned protein engineering procedures produced a high yield of soluble HERV-K Env SU for subsequent purification, crystallization, and crystallographic structure determination.

**Fig 1:**
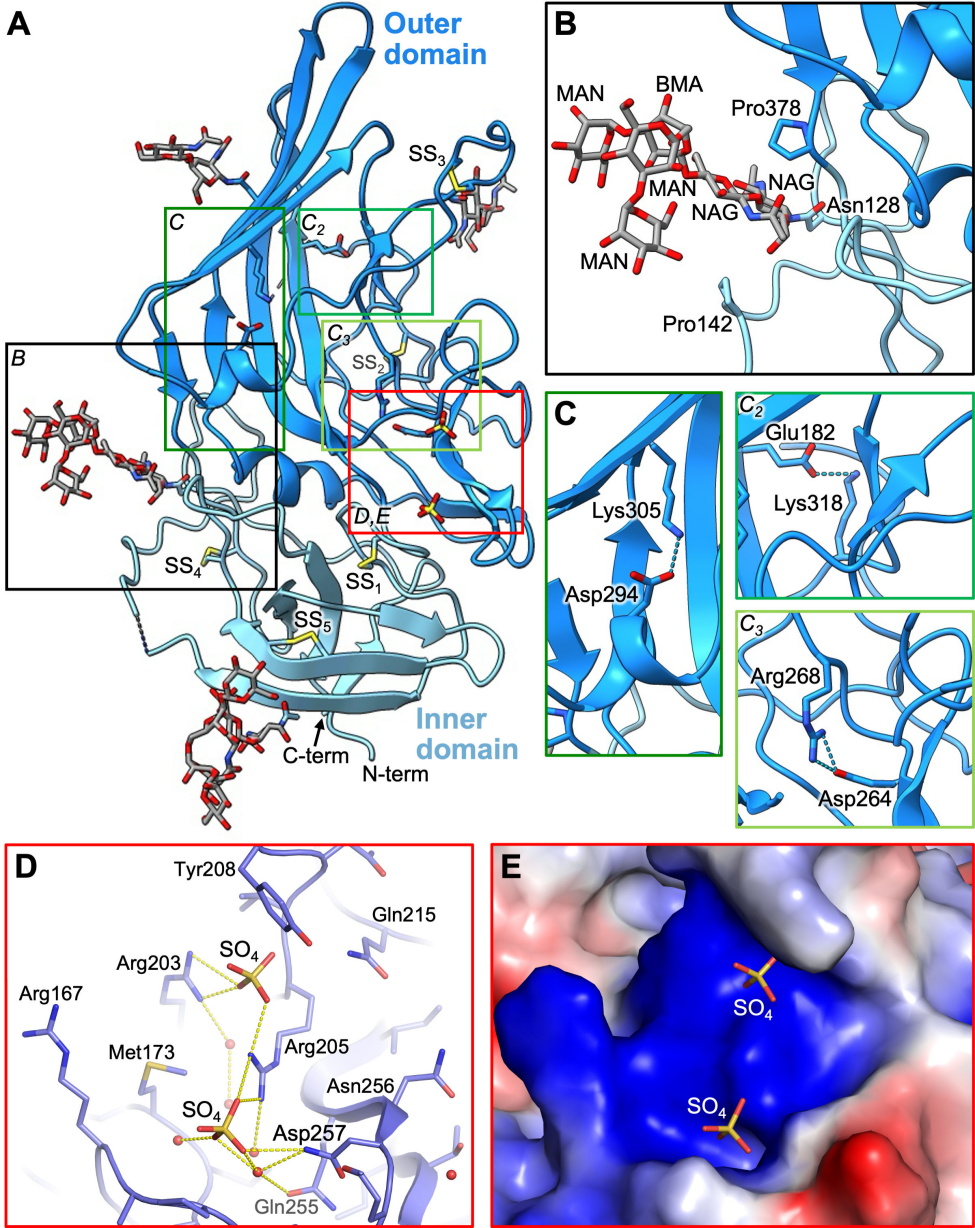
Overall fold and structural features of HERV-K Env SU. (**A**) Overall fold of (residues 111–430), consisting of inner and outer domains. Four N-linked glycans are shown in ball-and-stick representation with carbon atoms in gray. Five disulfides are shown in ball-and-stick representation with yellow sulfur atoms (SS_1-5_). The TM domain and viral membrane would be located below the N- and C-termini (N-term, C-term). (**B**) Closeup of the hexasaccharide glycan linked to Asn128 at the interface between the inner and outer domains. NAG, N-acetyl glucosamine; MAN, a-*D*-mannose; BMA, b-*D*-mannose. See Table 1 for crystallographic data collection, refinement, and validation statistics. (**C**) Closeups showing three salt bridges that stabilize the fold of the outer domain. (**D**) Closeup showing the two sulfate ions bound via salt bridges to Arg203 and Arg205. (**E**) Same view as in panel D with the protein in electrostatic surface representation (blue is positive, red is negative).

The crystal structure of HERV-K_CON_ Env SU was determined at 2.25-Å resolution by molecular replacement using an atomic search model generated with AlphaFold 3 (24). See Table 1 for crystallographic data collection, refinement, and validation statistics. The fold is composed primarily of β-sheets. An extended β-sheet network at the core of the protein provides structural stability and organizes the fold (Fig. 1A). The SU subunit can be subdivided into an inner and an outer domain, adopting terminology from the structure of the gp120 envelope glycoprotein from HIV-1 (25, 26). The inner domain (residues 111–171 and 379–430) forms a membrane-proximal structural unit that interfaces with the transmembrane (TM) subunit (21, 22), analogous to the interaction between HIV-1 gp120 and gp41 (the SU and TM subunits, respectively). The HERV-K SU inner domain consists of β-strands and loops and contains both the N- and C-termini of SU. The main structural feature of the inner domain is a distorted β-barrel of six β-strands, stabilized by three disulfide bonds, linking cysteines 164 and 171, 382 and 413, and 405 and 408. Notably, residues 405–408 are part of a CXXC motif, considered a hallmark of Envs from gamma-type retroviruses. The outer domain (residues 172–378) encompasses the expected receptor-binding region at the membrane-distal apex. It contains one long β-sheet of five antiparallel β-strands and one short β-sheet of three antiparallel β-strands, separated by two long loops, each stabilized by a disulfide bond (Cys227–Cys246 and Cys275–Cys282). An α-helix is also present near the interface with the inner domain.

**TABLE 1:** Crystallographic data collection, refinement, and validation statistics

Data collection	HERV-K Env SU^*a*^
Space group	*P* 1
Cell dimensions	
*a, b, c* (Å)	60.04, 63.52, 70.681
α, β, γ (°)	101.94, 101.825, 104.061
Resolution (Å)	59.32–2.25 (2.31–2.25)
Total reflections	162,480 (7,933)
Unique reflections	44,336 (3,144)
Multiplicity	3.7 (3.5)
Completeness	98.3% (96.6%)
*<I>* / s(*I*)	11.4 (0.7)
Wilson *B-*factor	47.2
*R*_merge_	0.114 (1.50)
*R*_pim_	0.070 (0.931)
CC1/2	0.979 (0.317)
Refinement and validation	
Resolution (Å)	59–2.25 (2.31–2.25)
No. reflections, working set	44,336 (3,144)
No. reflections, test set	2,016 (145)
*R*_work_	0.2126 (0.3597)
*R*_free_	0.2671 (0.3931)
No. non-hydrogen atoms	5,927
Protein	5,149
Ligands	486
Water	292
Root mean square deviations	
Bond lengths (Å)	0.008
Bond angles (°)	1.26
Ramachandran plot	
Favored (%)	96.22
Allowed (%)	3.78
Outliers (%)	0.00
Rotamer outliers (%)	1.2
ClashScore (Phenix 2.0)	8.64
Average *B*-factors^*b*^	61
Protein (Å^2^)	58
Ligands (Å^2^)	98
Solvent (Å^2^)	55
PDB accession code	9SYA

^*a*^ Numbers in parentheses refer to the highest resolution shell.

^*b*^ Residual B-factors after TLS refinement. See PDB entry for TLS parameters.

**Fig 2:**
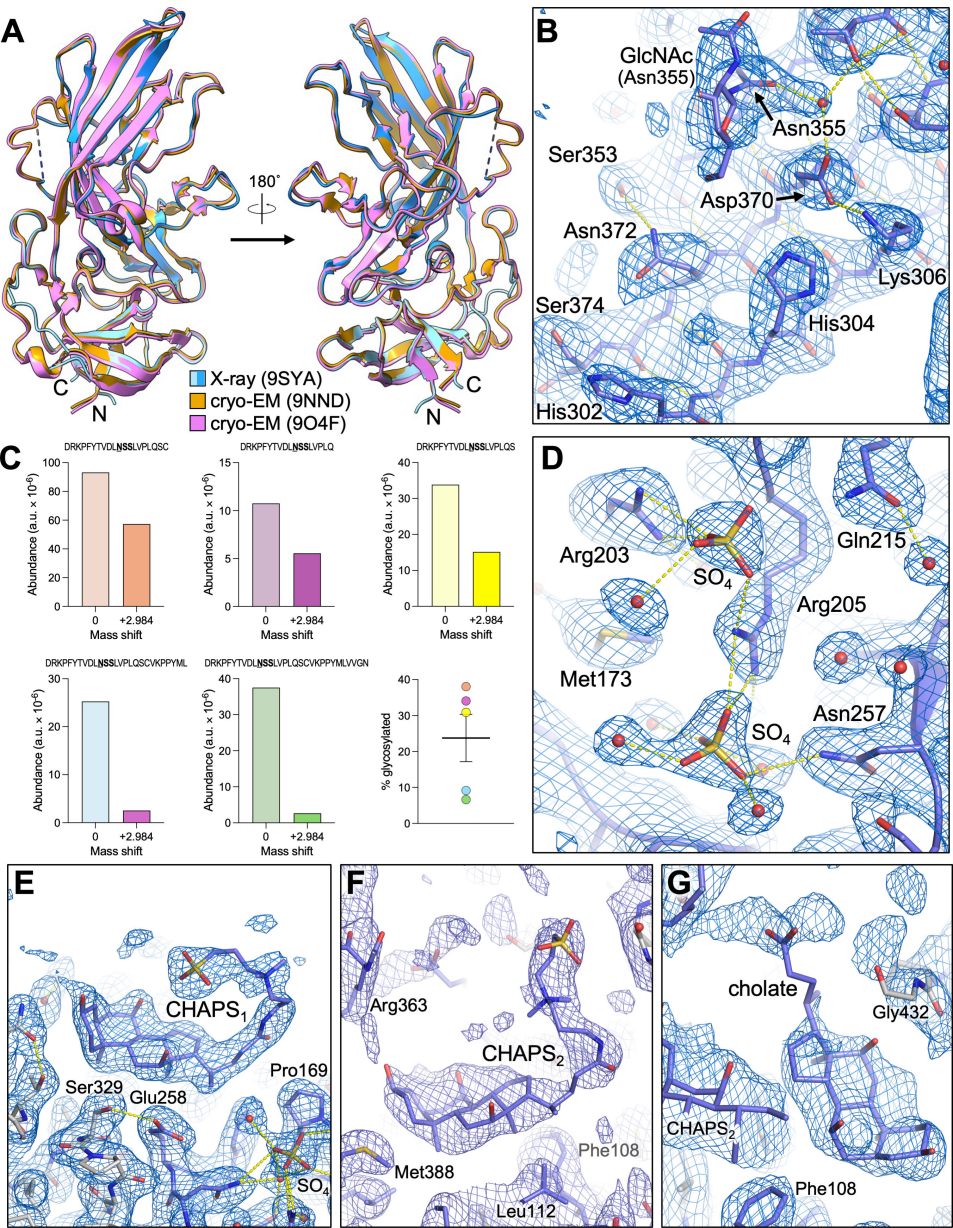
Analysis of the HERV-K SU structure and glycosylation status. (**A**) Superposition of the crystal structure of SU on cryo-EM structures of SU from two recent studies, PDB 9NND (21) and PDB 9O4F (22). (**B**) Electron density for Asn372 and surrounding region. The visible glycan is linked to Asn355. (**C**) Mass spectrometry of peptides containing Asn372 (N) treated with PNGase F in H_2_^18^O. N-glycosylated peptides acquired a +2.984 atomic mass shift. Peptide sequences and their abundances with and without the shift are shown. Bottom right, plot of the glycosylated fraction of each peptide using the same coloring scheme as in the histograms. The mass spectrometry source data are available in the supplementary material. (**D**) Electron density for sulfate ions. (**E–G**) 2*F*_O_ – *F*_C_ electron density maps for three cholic acid derivatives, contoured at 1 s with PyMol (Schrödinger, Inc.): (**E**) the first CHAPS molecule, (**F**) the second CHAPS molecule, and (**G**) the cholate molecule.

Comparison of the crystal structure of HERV-K SU with the cryo-EM structures of HERV-K Env shows that the SU domain has a very similar structure with root-mean-square deviation (RMSD) of Ca atoms of 0.33–0.56 Å. A few internal loops have slightly different conformations in the structure versus the cryo-EM structures (Fig. 2A). Electron density was present for four out of the five predicted N-linked sequons, at positions 128, 153, 274, and 355. At Asn128, the map contains clear density for the chitobiose core (two N-acetylglucosamines) plus four mannose moieties. The chitobiose core forms hydrophobic contacts with Pro378 in the outer domain (Fig. 1B). The glycan at position 153, although located in the inner domain, lacks the structure seen at position 128 but may nevertheless contribute more subtly to transient interactions with the TM subunit or modulate local folding dynamics. The glycans at positions 274 and 355 sit on the exposed outer domain and display more limited, peripheral density consistent with solvent-accessible, mobile oligomannose adducts that are unlikely to form a continuous steric cloak. There was no electron density for a glycan at Asn372 in the crystal structure (Fig. 2B), whereas an N-linked glycan was modeled at this residue in the cryo-EM structures (21, 22). However, mass spectrometry of SU peptides with isotope-coded glycosylation site-specific tagging (IGOT) showed that N-glycosylation was present at Asn372, although a substantial fraction of non-glycosylated peptides was also detected (Fig. 2C). This suggests that the lack of glycan density at Asn372 is due to either conformational flexibility of the glycan or preferential crystallization of the protein fraction lacking glycosylation at Asn372. Together, these observations define a spatially sparse glycan landscape on HERV-K SU that contrasts sharply with the dense glycan shield of HIV-1 gp120 (25–27) and more closely resembles the lighter glycosylation reported for Syncytin-2 SU subunit (28). The architecture implies relatively large protein surfaces will be available in HERV-K SU for receptor engagement and antibody recognition, with the glycan at Asn128 representing a localized structural exception rather than part of an immune-evasive glycan cloak.

Within the outer domain, two salt bridges play key roles in structural stabilization. The first, between Asp264 and Arg268, reinforces the short β-sheet of 3 antiparallel β-strands and its adjoining loop (Fig. 1C). The second, between Asp294, located within a short α-helical segment, and Lys305, stabilizes the longer five-stranded β-sheet together with the adjoining extended loop region (Fig. 1C). Together, these interactions delimit and stabilize the surface-exposed loop. A third salt bridge is present between Glu182 of the longer β-sheet and Lys318 of a smaller loop between the two β-sheets (Fig. 1C). Other notable structural features are three relatively rare *cis* peptide bonds involving residues Gly135–Pro136, Ser329–Pro330, and Pro385–Pro386.

### Sulfate ions bind to a putative heparan sulfate binding site

Each protein subunit in the crystal structure was modeled with a pair of sulfate ions, which are coordinated via salt bridges by two arginine residues, Arg203 and Arg205. The sulfate ions bind in pairs to a shallow groove on the surface of SU (Fig. 1D, E and 2D). HERV-K SU was previously shown to bind heparan sulfate, and attachment to cell-surface heparan sulfate was required for cell entry of virions pseudotyped with HERV-K Env (19). Arg203 and Arg205 contribute to a shallow groove with a strongly positive charge on the surface of HERV-K Env. Shaked et al. noted that Arg203 and Arg205 are conserved and the groove they form could accommodate a segment of heparan sulfate polymer with up to three negatively charged sulfate groups (21). Electron density for the sulfate ions was relatively weak in the refined crystal structure, consistent with partial (50%–60%) occupancy. Since the crystallization buffer contained sulfate, phosphate, and nitrate (30 mM each), it is possible that a mixture of these three anions accounts for the observed electron density. Nevertheless, the presence of two polyatomic anions coordinated by Arg203 and Arg205 is entirely consistent with the binding of heparan sulfate at this site.

### Cholate molecules bind hydrophobic surfaces at the TM interaction interface

**Fig 3:**
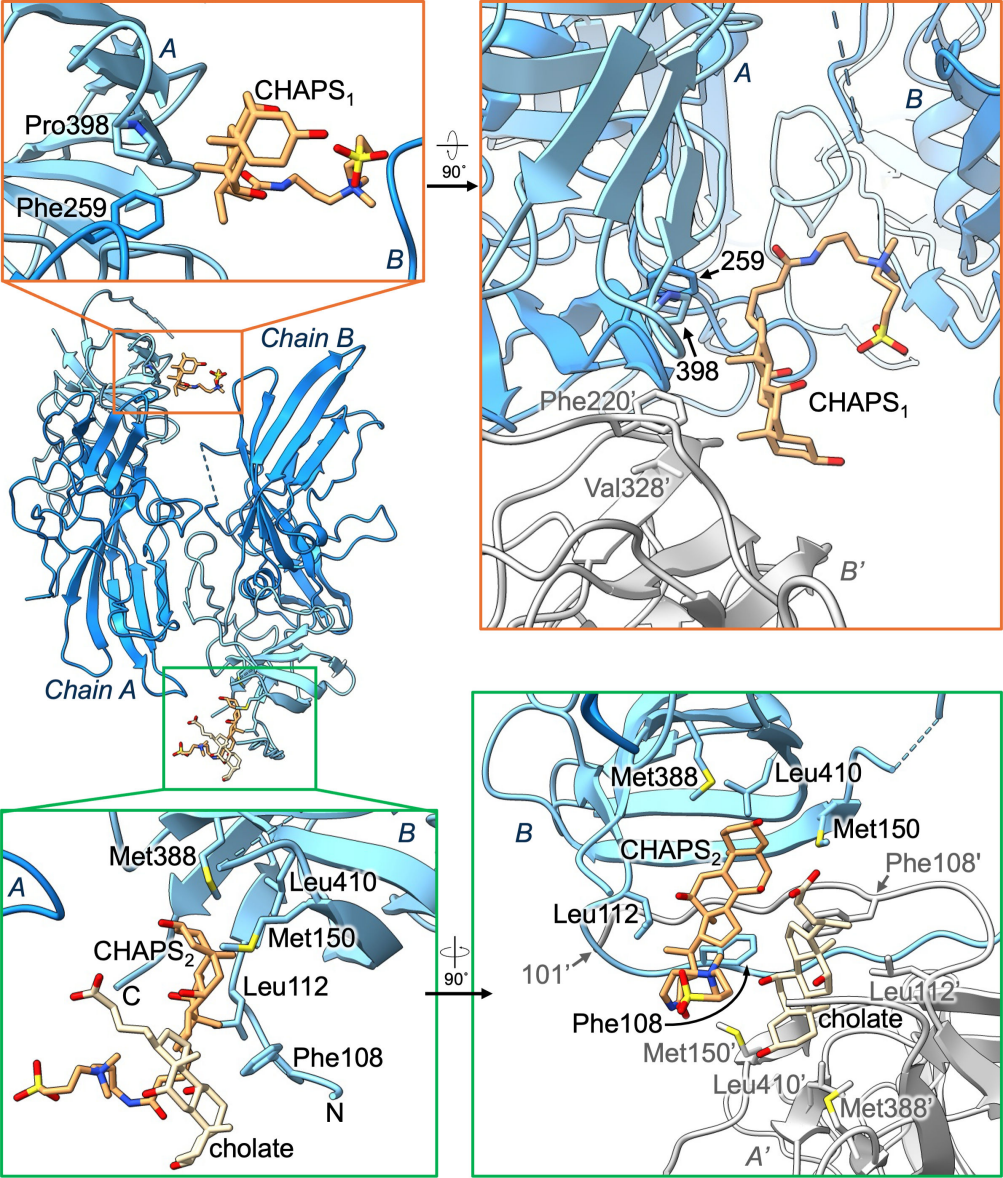
Binding mode of three sterol molecules to HERV-K SU. Three cholic acid (cholate) derivative molecules were clearly identifiable in the electron density map. Two were modeled as CHAPS (3-[(3-cholamidopropyl)dimethylammonio]−1-propanesulfonate) and one as cholate. Cholic acid derivatives were present as additives in the crystallization condition (see Materials and Methods) (29). CHAPS and cholate molecules are shown in ball-and-stick representation with yellow carbon atoms. “*A”* and “*B”* denote two HERV-K SU chains in the crystallographic asymmetric unit. Proteins shown in gray are related to the asymmetric unit by crystallographic symmetry. Their chain and residue identifiers are shown in gray and followed by a prime symbol.

A molecule of CHAPS (3-[(3-cholamidopropyl)dimethylammonio]−1-propanesulfonate), a cholate (cholic acid) derivative included in the crystallization solution (29), was modeled within a hydrophobic pocket at the interface between the outer and inner SU domains (Fig. 2E and 3). The cholate four-ring steroid nucleus—specifically rings C and D and one protruding methyl group on its convex face—nestles into this pocket, which is formed primarily by Phe259 (outer domain) and Pro398 (inner domain).

A second CHAPS molecule and a cholate molecule were modeled on concave hydrophobic surface patches on the inner SU domain adjacent to the trypsin-derived signal-peptide tail (Fig. 2F, G and 3). This CHAPS molecule makes hydrophobic contacts with a hydrophobic surface clusters formed by Met150, Met388, Leu112, and Leu410, along with a phenylalanine from the trypsin-derived signal-peptide tail (residues 100–110; Fig. 3). The cholate molecule is stabilized by residues from an adjacent SU molecule in the crystal lattice (crystallographic symmetry packing interactions), as well as by interactions with the neighboring CHAPS molecule. These cholate-containing ligands stabilize structural features in the SU fragment that in the intact envelope complex are maintained via interactions with the TM subunit (21, 22).

### Comparison to other retroviral env SU structures

**Fig 4:**
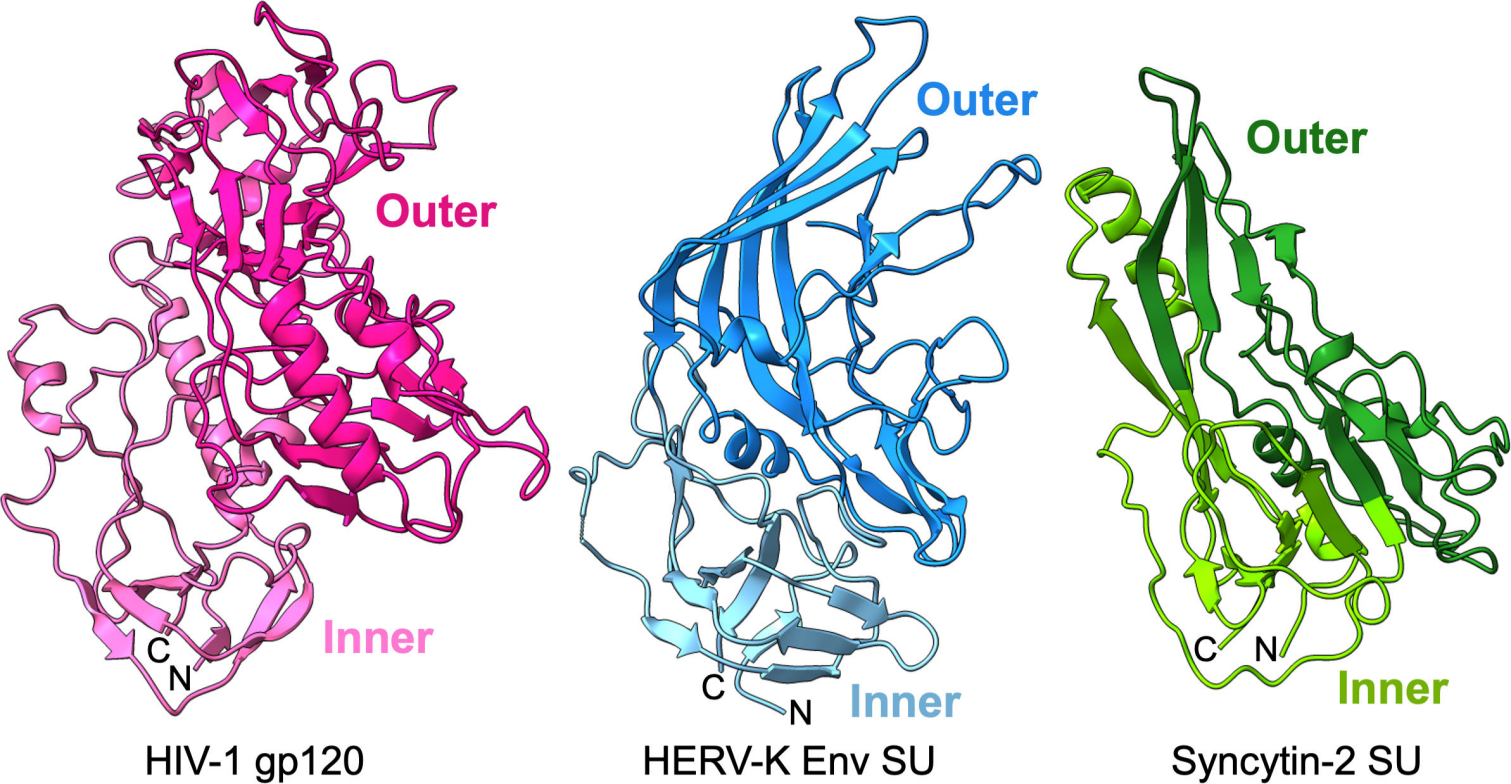
Comparison of HERV-K Env SU to Syncytin-2 SU and HIV-1 gp120. Structures of HIV-1 gp120 (PDB 6PWU [27]), left, and Syncytin-2 SU (PDB 7OIX [28]), right, the closest structural homologs of HERV-K SU. The relative orientations of the proteins reflect structural alignment of their inner domain using the HERV-K SU inner domain as the reference. The root mean square deviation of the inner domain Ca positions after superposition onto the HERV-K SU inner domain was 2.89 Å for Syncytin-2 SU and 4.01 Å for HIV-1 gp120. The N- and C-termini of each SU domain are labeled N and C, respectively.

A structural similarity search with DALI (30) identified Syncytin-2 SU as the protein with the most similar structure to HERV-K SU (Z-score 7.5, Rmsd 5.9 Å, PDB 7OIX [28]). The next most similar structures were those of HIV-1 gp120 (Z-score 3.2, Rmsd 5.4 Å, PDB 6PWU [27]) and SIV gp120 (Z-score 2.8, Rmsd 3.4 Å, PDB 8DUA [31]), although the similarity scores for these were only marginally higher than for clearly unrelated proteins (Z-score 2.7 and below). Syncytin-2, which drives cell-cell fusion of trophoblasts fusion in placental development, is derived from the Env of HERV-FRD, a gammaretrovirus-like endogenous retrovirus (28, 32, 33). Most of the structural similarity between HERV-K SU, Syncytin-2 SU, and HIV-1 gp120 maps to the inner domain, which has a similar distorted β-barrel as its core architectural element in all three proteins (Fig. 4). Upon superposition on HERV-K SU inner domain, the root mean square deviations of the inner domain Ca positions of Syncytin-2 SU and HIV-1 gp120 were 2.89 Å and 4.01 Å, respectively. Given that the inner domain is proximal to the TM membrane fusion subunit, the structural conservation of this domain suggests a common mode of intramolecular communication within the heterodimeric Env complexes.

The outer domain of HERV-K SU incorporates a mixed architecture comprising β-sheets, loop insertions, and helical elements. The main structural feature is a long β-sheet composed of five antiparallel β-strands, a topological element also found in the outer domain of Syncytin-2 SU. However, the structures of the outer domains, and the insertions within them, are otherwise highly divergent in HERV-K SU, Syncytin-2 SU and HIV-1 gp120 (Fig. 4).

## DISCUSSION

Here, we report the crystal structure and associated atomic model of the envelope glycoprotein Surface (SU) subunit from the human endogenous betaretrovirus HERV-K (HML-2). The HERV-K SU fold retains the two-domain topology, with inner and outer domains, seen in other retroviral SU subunits including gammaretroviruses (Syncytin-2) and lentiviruses (HIV-1). This topological conservation demonstrates that a common architectural blueprint underlies Env proteins across the *Orthoretrovirinae* subfamily of *Retroviridae* (which includes the genera alpharetrovirus, betaretrovirus, gammaretrovirus, lentivirus, and others), with genus-specific refinements (glycan decorations, loop insertions, receptor specificities) overlaying the common framework. However, despite the similarity in secondary structure topology to other retroviral SUs, perhaps the most striking feature of the structure of HERV-K SU is how different its three-dimensional structure is from those of its closest structural homologs, Syncytin-2 SU and HIV-1 gp120. This indicates that HERV-K SU has diverged further from the SUs of other gamma- and gamma-like retroviruses at the structural level than might have been expected from phylogenetic analysis.

The crystal structure of HERV-K SU, determined at 2.25-Å resolution, reveals various HERV-K-specific structural elements. The fold is stabilized by five disulfide bonds and three key salt bridges. Two loop regions provide an extensive surface for potential interactions with cell-surface receptors or other cellular factors. The structure offers a detailed view of the glycan landscape. The N-linked glycans provide relatively sparse coverage of the molecular surface of SU, standing in contrast with the dense glycan shield of HIV-1 gp120. The crystal structure also contains three amphiphilic steroid molecules (cholate or cholate derivatives), similar in structure to cholesterol, bound to hydrophobic surface patches of SU. These could prove to be attractive starting points for small-molecule or fragment screening campaigns to identify compounds that bind to SU and interfere with the membrane fusion activity of HERV-K Env or its ability to interact with other cellular proteins.

The HERV-K SU crystal structure reveals the conserved structural features shared with other orthoretroviral Envs while also identifying HERV-K-specific features, namely its sparse glycocalyx, bound sulfate ions, hydrophobic surface patches, and extended surface-exposed loops. The sulfate ion-binding sites reveal the putative location of the heparan sulfate binding on HERV-K Env, reported to be important for cellular attachment and infection (19). Autoantibodies against HERV-K Env have been reported in patients with various autoimmune diseases (16, 17). Given that SU is the receptor-binding subunit in retroviruses and contains most of the known antibody-binding epitopes, the epitopes of disease-causing autoantibodies against HERV-K Env are likely to map to the solvent accessible surface of SU. The structure of SU will be an invaluable tool in future studies to identify these epitopes. More broadly, the availability of a high-resolution crystal structure for HERV-K SU, along with our optimized expression construct and purification protocol, open paths toward extending our mechanistic understanding of betaretroviral envelope proteins and developing translational applications targeting HERV-K elements, the most active and recently acquired endogenous retroviruses in humans.

## MATERIALS AND METHODS

### Cloning of expression vectors

The gene encoding the SU domain of HERV-K_CON_ Env (residues 111–430) (7) was cloned into the pcDNA3.1(+) plasmid. To enhance protein secretion, an N-terminal signal peptide–pro-peptide domain derived from human Trypsin-1 was fused to the gene (23), followed by a human rhinovirus (HRV) 3C protease cleavage site. A C-terminal hexahistidine (His6) tag was added for purification purposes, preceded by a Pro-Gly sequence to facilitate efficient removal of the His6 tag by carboxypeptidase A. Additionally, the sequence was engineered to substitute cysteine at position 141 with alanine to prevent oligomerization caused by the free cysteine residue.

### Protein expression and purification

The HERV-K Env SU domain was expressed in HEK293S GnTI^−^ cells adapted for suspension culture and maintained at 37°C in a humidified atmosphere containing 8% CO_2_ with shaking. Cells were grown in serum-free Expi293 Expression Medium (Gibco) to a final density of 3 × 10⁶ viable cells/mL, with 95%–99% viability before transfection. Transfection was performed using 1 mg/mL polyethylenimine (PEI) at a DNA:PEI ratio of 1:3 (w/w). Secreted protein was harvested 5 days post-transfection by collecting the supernatant after centrifugation at 300 × *g* for 5 min at 4°C to pellet cells. The clarified medium was applied to Ni Sepharose Excel resin (Cytiva), washed, and eluted with 50 mM Tris pH 7.4, 300 mM NaCl containing 10 and 250 mM imidazole, respectively. The protein was further purified by size-exclusion chromatography using a Superdex 200 Increase 10/300 GL column (Cytiva) equilibrated in 50 mM Tris pH 7.4, 100 mM NaCl.

### Crystallographic structure determination

Crystals were grown at 18°C by sitting-drop vapor diffusion. Purified protein was concentrated to 11.7 mg/mL, and 1 volume of seed stock was added to 5 volumes of protein solution. The final solution was mixed with an equal volume of reservoir solution optimized from the Morpheus Fusion Screen (Molecular Dimensions) (29): 32.7% PEG 8K/ethylene glycol (1:2), 0.1 M MES/imidazole pH 6.5, 90 mM Morpheus NPS, and 1.572% (w/v) Morpheus III cholic acids. Seed stock was prepared by crushing small crystals from a previous optimized crystallization experiment conducted with varying PEG 8K/ethylene glycol (1:2) concentrations (18%–36%) and Morpheus III cholic acids (0.24%–1.8% w/v), while maintaining constant 0.1 M MES/imidazole pH 6.5 and 90 mM Morpheus NPS (nitrate phosphate sulfate). X-ray diffraction data were collected at 100 K using Pilatus3 6M and Eiger2 XE 16M detectors at Diamond Light Source beamlines I24 and I04, respectively. Data were processed with autoPROC v1.0.5 (34) and Xia2 (Dials, Aimless) (35). Molecular replacement was performed with Phaser (36) as implemented in PHENIX v1.21.1 (37) using an atomic search model of the SU domain of HERV-K_CON_ Env from AlphaFold 3 (24). An initial model was built using AutoBuild in PHENIX, manually completed with COOT v.0.9.6 (38), and iteratively refined with PHENIX v2.0. See Table 1 for crystallographic data collection, refinement, and validation statistics.

### Mass spectrometry with isotope-coded glycosylation site-specific tagging (IGOT)

Glycopeptides were extracted from SDS-PAGE gel bands by digestion with trypsin or Asp-N protease. The glycopeptides were treated with Peptide-N-glycosidase F (PNGase F) in the presence of heavy water (H_2_^18^O). If an N-linked glycan is present, PNGase F removes it and converts the linking asparagine residue to aspartate. During this conversion, ^18^O is incorporated into the peptide, resulting in a net atomic mass shift of +2.984. The labeled peptides were analyzed by liquid chromatography-tandem mass spectrometry (LC-MS/MS) on an Orbitrap Eclipse mass spectrometer couple to a Vanquish Neo nHPLC (Thermo Fisher Scientific). Raw data were imported and data processed in Proteome Discoverer v3.1 (Thermo Fisher Scientific). A spreadsheet containing the mass spectrometry data from which the histograms in Fig. 2C were calculated is available in the supplementary material.

### Structural analysis

Coordinate files were submitted to PDBsum (39) for structural feature analysis. Molecular visualization and figure generation were performed using UCSF ChimeraX (40) and PyMOL (Schrödinger, Inc.). Structural homologs were identified using the DALI server (41), and 3D structural comparisons were carried out using the TM-align algorithm (42).

## Data Availability

The atomic coordinates of the HERV-K SU glycoprotein fragment were deposited in the Protein Data Bank with accession code 9SYA (https://doi.org/10.2210/pdb9sya/pdb).

## SUPPLEMENTAL MATERIAL

Supplemental material jvi.00195-26-s0001.xlsx Liquid chromatography-tandem mass spectrometry (LC-MS/MS) source data.

ASM does not own the copyrights to Supplemental Material that may be linked to, or accessed through, an article. The authors have granted ASM a non-exclusive, world-wide license to publish the Supplemental Material files. Please contact the corresponding author directly for reuse.
